# Meningeal Tuberculoma Masquerading as a Brain Tumor: A Case Report

**DOI:** 10.7759/cureus.30804

**Published:** 2022-10-28

**Authors:** Mohammad Abu-Abaa, Hassaan Arshad, Doaa Ali, Ali Abdulsahib

**Affiliations:** 1 Internal Medicine, Capital Health Regional Medical Center, Trenton, USA

**Keywords:** hiv-positive, brain tumor, meningeal tuberculous, intracranial tuberculoma, central nervous system tuberculosis

## Abstract

Central nervous system (CNS) tuberculosis (TB) is a common extrapulmonary manifestation of TB. However, tuberculoma is a rare finding and meningeal tuberculoma is even rarer. This is a case report of a 47-year-old recent immigrant from Africa who presented with stroke-like symptoms. The human immunodeficiency virus (HIV) screening was reactive. Imaging revealed significant vasogenic edema surrounding a brain mass. Biopsy proved TB, and symptoms improved with steroids and anti-TB medication. This case serves to remind clinicians of a rare form of TB that can mimic brain tumors and strokes in presentation.

## Introduction

Tuberculoma is an intracranial process of granulomatous tuberculosis tissue contained by the host’s immune response. Central nervous system (CNS) tuberculosis (TB) is seen only in 10% to 20% of those with extrapulmonary TB and tuberculomas are seen in only 1% [1]. It usually results from hematogenous spread from a pulmonary focus. However, no pulmonary infection is seen in 25% to 30% of CNS TB [2]. This case aims to highlight this rare form of TB and its presentation. 

## Case presentation

**Figure 1:**
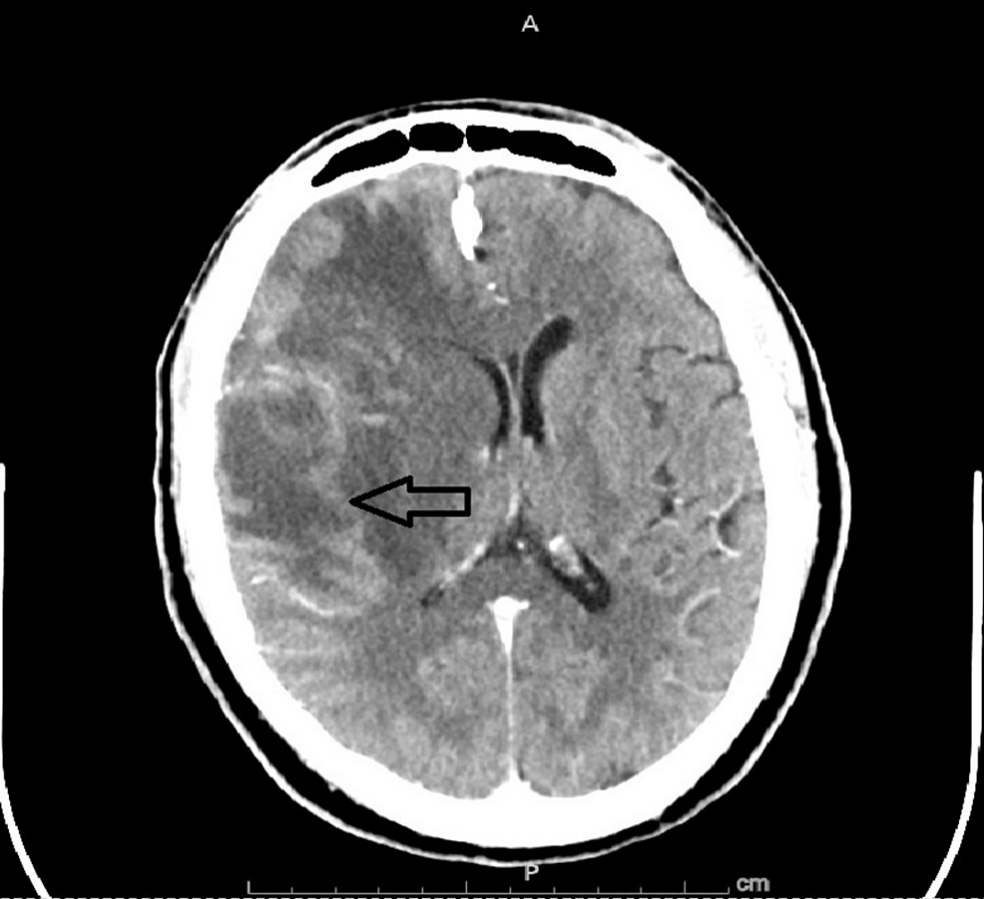
Initial CT of the head The CT of the patient's head shows a large right sided, mainly peripherally enhancing mass surrounded by large vasogenic edema (arrow).

A 47-year-old male patient who is a recent immigrant from Africa and has been in the US for two years presented to the emergency department (ED) with a history of falling off the bed with head and neck trauma six days before presentation with intermittent left-sided headache and neck pain. He also was complaining of dizziness and left-sided weakness for six days. His wife reported him dragging his left foot and dropping objects held by his left hand. Past medical history was unremarkable except for a prior gunshot wound and residual bullet fragments. In the ED, a physical exam showed a left-sided facial droop with muscle weakness 4+/5 on the left upper and lower extremities. Pupils were 3 mm equal and symmetrical bilaterally. Otherwise, the physical exam was unremarkable. Vital signs were within normal limits. Basic labs were unremarkable. Computed tomography (CT) scan of the head with contrast initially showed a large 3.9 cm poorly defined and predominantly enhanced right-sided frontoparietal mass with surrounding vasogenic edema with calcifications and petechial hemorrhages. Significant effacement and mass effect on the right lateral ventricle with an 8 mm midline shift was also noted (Figure 1). Chest X-ray was unremarkable for any opacity. The patient was started on steroids and prophylactic levetiracetam and admitted to the neuro ICU.

The CT of the chest, abdomen, and pelvis were unremarkable for any mass. More delineation by MRI of the abdomen was not feasible due to the history of gunshot wound to the right hip with a lingering bullet slug. The human immunodeficiency virus (HIV)-1 screening test was positive which was raising suspicion about the infectious nature of the mass. Viral load was 14000 copies/ml and clusters of differentiation (CD)+4 cell count was at 16 cells/ml. Further history revealed a distant history of TB infection while in Africa. Cerebrospinal fluid (CSF) analysis showed mild lymphocytic pleocytosis at 16 cells/ml and elevated protein at 149 mg/dl and normal glucose at 49 mg/dl. The CSF culture remained negative and the Quantiferon release assay was indeterminate. Toxoplasma immunoglobulin (Ig)G was positive in the CSF, for which the patient was started on trimethoprim-sulfamethoxazole. The CSF was negative for cryptococcal, Epstein Barr virus (EBV), cytomegalovirus (CMV), and venereal disease research lab (VDRL) test. The CSF acid-fast staining was negative even after one week of incubation. Meningitis/encephalitis panel including *Cryptococcus*, varicella-zoster virus (VZV), *Streptococcus*, *Neisseria*, *Listeria*, human *Parechovirus*, human herpes virus 6, human simplex virus type 1 and 2, *Haemophilus influenzae*, *Escherichia coli* K1, *Enteroviru*s, and *Cytomegalovirus* were also negative. The CSF flow cytometry was negative for B-cells ruling out lymphoma.

Seven days into hospitalization, the patient's neurological status deteriorated with progressive weakness on the left side 0/5 and intermittent confusion. Pupils remained symmetrical and reactive bilaterally. He was started on continuous electroencephalogram (EEG) monitoring and mannitol and hypertonic saline 3%. A repeat CT head with contrast showed no change in the size of the mass. He clinically improved with increasing steroid, mannitol, and hypertonic saline to ⅗ muscle power on the left side. The EEG was negative for ictal activity. A craniotomy and brain biopsy was performed. Craniotomy for brain biopsy was done and the sample was sent to two tertiary care centers for double confirmation of the results which showed extensive multifocal perivascular and angioinvasive lymphoplasmacytic infiltration suggesting vasculitis. Inflammatory infiltrates also showed B and T cells with macrophages. Clonal T cells were also noted by polymerase chain reaction (PCR) analysis. Staining for fungi and toxoplasma was negative. However, acid-fast bacilli (AFB) staining was positive, establishing the diagnosis of tuberculoma. He was started on isoniazid, ethambutol, pyrazinamide, rifampin and vitamin B6.

**Figure 2:**
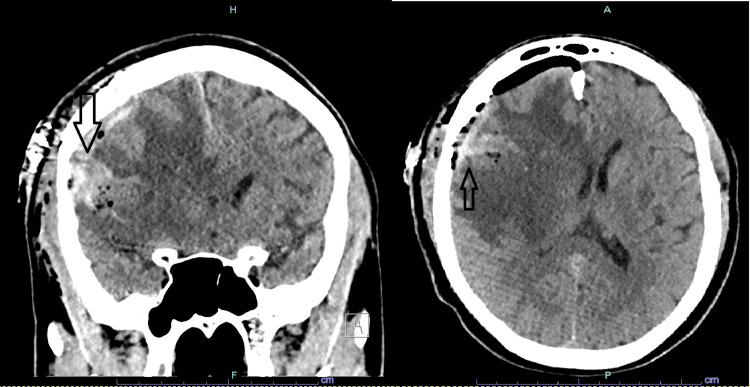
The post-craniotomy CT of the head shows a meningeal origin of the mass in both the coronal and transverse sections.

The CT head post-craniotomy and weeks of anti-TB regimen showed a reduction of the vasogenic edema and allowed more delineation of the mass. Coronal and transverse CT sections showed the mass originating from the meninges (Figure 2). The patient was also started on emtricitabine, tenofovir, and dolutegravir combination. Clinical improvement allowed for discharge on the anti-TB regimen along with an anti-HIV regimen. Anti-epileptic medication was also continued as an ictal activity was noticed clinically and on EEG post-craniotomy. A clinical follow-up six months after discharge showed no clinical neurological deficit.

## Discussion

Meningeal tuberculoma is a rare entity in developed countries with an incidence rate of 0.2% and is more commonly seen in developing ones, accounting for 5% to 10% of all intracranial masses [3]. It is more commonly seen in young age patients [3] like ours where it usually presents in immunocompromised individuals [4]. The risk is five times higher for HIV-positive patients [5]. The most common locations are the periventricular gray-white matter junction and posterior fossa. Meningeal-based lesions are rare [6]. Dural-based lesions or parenchymal lesions that are believed to be originating from dura are termed pachymeningeal TB [7]. 

Pathogenesis has been described in two stages. First is the development of Rich’s lesions during the period of bacteremia or shortly afterward, and the second is the rupture of these lesions. Rich’s lesions can be seen in meninges or subpial or subependymal spaces and may be dormant for several years. The trigger for rupture is unknown but likely immunological in nature. Immunological status and bacterial as well as host genotype determine the extent of extrapulmonary spread [8]. In our patient, his immune compromise likely contributed to the development of tuberculoma. 

As in our patient, tuberculoma usually presents with progressive focal neurological deficits. Altered sensorium is also possible usually in the setting of fever and headache. Lack of pulmonary lesions/symptoms does not preclude the diagnosis. In this case, diffusion and spectroscopy are useful to differentiate tuberculomas from brain tumors and other infectious etiologies. On an enhanced CT scan, the target sign is pathognomonic and is characterized by central hypodensity and ring-like enhancement. Enhancement on MRI is seen in caseating lesions. It is usually solitary and multiple lesions are more suggestive of tuberculosis and usually tend to affect the frontal and parietal areas, as seen in our patient [8]. As seen in our case with isolated CNS tuberculoma and parenchyma involvement, CSF analysis may be normal if isolated CNS tuberculoma, and CSF culture may be negative if brain parenchyma is involved. The CSF PCR is useful but time-consuming [2]. The diagnosis remains dependent on biopsy findings [3]. It usually shows caseating granulomas but non-caseating ones can be seen in 14% of cases [9]. 

Medical management is usually pursued with a four-drug regimen with a variable recommended duration of six to nine months or 18 months [6]. Craniotomy for brain biopsy is also needed for a definitive diagnosis when diagnostic uncertainty exists. A large placebo-controlled trial showed improved mortality with the addition of dexamethasone which reduces the adverse immunological reactions, and incidence of hydrocephalus and strokes [10]. Dexamethasone is believed to reduce brain and vascular inflammation and thus reduce intracranial pressure. Paradoxical enlargement or development of tuberculoma on anti-TB medications has been described and is likely immunological in nature [11]. Resolution can be expected in 78% of cases within one to two years [2].

## Conclusions

Meningeal tuberculoma is a rare entity. It is more commonly seen in young age patients and immunocompromised individuals. It usually results from hematogenous spread from pulmonary TB. Tuberculoma usually presents with acute or subacute onset of progressive focal neurological deficits. It is difficult to differentiate between tuberculoma from brain tumors especially primary CNS lymphoma in HIV-positive patients. Thus, brain biopsy is usually necessary and holds a superior diagnostic value than CSF analysis. This case highlights the importance of maintaining a high clinical suspicion of TB and pursuing extensive history-taking, especially in the context of immunocompromised patients and recent immigration. 
